# Modulation of antibody secreting cells and neutralizing Ab activity in HIV infected individuals undergoing structured treatment interruptions

**DOI:** 10.1186/1742-4690-9-S2-P63

**Published:** 2012-09-13

**Authors:** A Llano, J Carrillo, B Mothe, S Marfil, E García, L Ruiz, E Yuste, V Sanchez, J Blanco, C Brander

**Affiliations:** 1 IrsiCaixa AIDS Research Institute, Barcelona, Spain; 2 Institut d'Investigacions Biomediques August Pi i Sunter, Barcelona, Spain

## Background

HIV-1 infection generates numerous abnormalities in the B cell population. The majority of these defects are reverted by antiretroviral therapy. Our aim was to evaluate the effects of re-exposure to HIV antigens on the frequency and functionality of antibody secreting cells (ASC) in patients undergoing structured treatment interruptions (STI). As re-exposure to viral antigens may also boost the production of (neutralizing) antibodies, we also assessed the neutralizing activities during STI cycles.

## Methods

Retrospective study of 10 patients undergoing 3 cycles of STI with 2 weeks on and 4 weeks off HAART. ASC frequencies were determined by flow cytometry in samples obtained at the beginning and the end of STI. Neutralization capacity, total IgG concentration and anti-gp120-IgG titres were evaluated.

## Results

Median viral loads were higher at the end of STI compared to time of treatment stop: 20[20-1200] vs 615[20-452000] respectively for the first STI, 20[20-654] vs 3655[20-45900] (p<0.05) for the second and 35[20-82] vs 290[20-17400] (p<0.05) for the third STI. The frequency of ASC followed the same trend: 0.35%[0.17-1.15] at the beginning of first STI vs 0.27[0.20-4.95] at treatment restart, 0.35[0.10-1.40] vs 0.82[0.30-3.25] for the second and 0.30[0.10-0.70] vs 0.40[0.15-1.85] for the third STI. Eight out of 10 patients maintained stable total IgG levels during the study. HIV-neutralizing activity was observed in two patients concomitantly with high anti-gp120 titters. In one patient the neutralizing activity remained constant while the second patient showed elevated neutralizing Ab after first STI and once treatment was reinitiated after the 2nd STI.

## Conclusion

Our data suggest that STI and its associated transient increases in viral load drive the frequencies of ASC in an antigen-specific manner. In some subjects, this re-exposure to autologous virus boosts the presence of neutralizing antibodies, albeit in a somewhat delayed manner.

